# Book review: “Epigenetics”

**DOI:** 10.3389/fgene.2014.00104

**Published:** 2014-04-30

**Authors:** Steven G. Gray

**Affiliations:** Department of Clinical Medicine, St. James Hospital/Trinity College DublinDublin, Ireland

**Keywords:** epigenetics, introductory textbook, primer, cellular functions, disease

The field of Epigenetics is rapidly evolving, such that the pace of our advances in the knowledge and understanding of this phenomenon often outstrips the basic teaching literature. In the preface to this book, the author describes the rationale for the book was to develop a text that would provide an “easily digestible text that could provide an introduction to the subject of epigentics.” In essence, this is what this book does provide, as it is an easily read and understandable piece of work which introduces the novice to the world of epigenetics.

The book is split over 16 chapters into three main areas or sections. The first section deals with the general issues on how epigenetic regulation/modification is achieved with an introduction to basic chromatin structure and key concepts relating to the issues of regulating transcription, followed by how this regulation can be achieved through the auspices of the epigenetic machinery. There are eight chapters associated with this section dealing with various elements including chapters on chromatin (Chapters 3 and 4), DNA methylation (Chapter 5), Histone post-translational modifications (Chapter 6), and on Locus-specific control of chromatin structure (Chapter 8).

Having covered this, the reader is then brought into the second section of the book which deals with how epigenetics controls cellular functions, with five specific chapters on issues such as cell-specific gene expression (Chapter 9), the role of epigenetics in the regulation of the mitotic cell cycle (Chapter 10), the basis of genomic imprinting (Chapter 11, and an absolute requisite chapter for a book on epigenetics), through the role of epigenetics in cellular differentiation and reprogramming. In this regard the book is well represented as this is the authors area of speciality, and the chapters entitled “Epigenetic Control of Cellular Differentiation” (Chapter 12) and “Reversibility of Epigenetic Modification Patterns” (Chapter 13) nicely discuss the role of epigenetics and the potential clinical implications of aberrant epigenetics in both cellular reprogramming and induced pluripotent stem cell (iPSC) production.

The final section has three chapters discussing the evidence for the various roles of epigenetics with disease development and progression. The main critique of this section is the paucity of chapters. There are only three chapters linking the wealth of information now linking epigenetics with disease. Chapter 14 is entitled “epigenetic predisposition to disease and imprinting based disorders.” The author briefly touches on predisposition briefly mentioning epidemiology and the potential role of epigenetics as a cause of stochastic variation in disease. Imprinting based disorders are discussed in more detail with emphasis on some of the “classical” examples of such disorders (Prader-Willi and Angelman syndrome) followed by Beckwith-Wiedemann and Silver-Russell syndromes. The areas dealing with epigenetics of major disease groups is in fact fairly limited and focuses primarily on cardiovascular disease with smaller texts on kidney disease and diabetes, with some of the evidence for epigenetics simply described for each. Admittedly in this chapter summary the author notes this limitation. Chapter 15 deals with the roles of epigenetics within the neuronal setting. The introductory sections dealing with the roles of epigenetics and memory formation is nicely written. Two examples of the role of epigenetics in neurodegeneration are discussed (Alzheimers and Parkinson's disease), and the potential role of epigenetics in mental health is discussed fairly superficially in relation to bipolar disorders and depression. While there is some discussion on the effects of cocaine on epigenetics in relation to depression, there is no discussion on how epigenetics may play roles in drug addiction. The final chapter deals with “Epigenetics of Cancer” (Chapter 16), a daunting task for any author. Not unexpectedly, this chapter was unfortunately very superficial discussing in general terms the roles of aberrant DNA CpG Methylation and histone post-translational modification patterns in cancer. There was some discussion of the role of miRNAs in regulating DNA methyltransferases but no real discussion on the roles of miRNAs and lncRNAs in cancer. There was no chapter on the available data relating to the discovery and development of the currently licensed FDA approved epigenetic targeting agents and the current excitement within the field moving forward with the novel agents currently being developed for various epigenetic “readers,” “writers,” and “erasers.”

Nevertheless, the book itself is well written, easy to read and more importantly easily understood. There are elements of this book which shine, and conversely elements that grate, particularly for those familiar or expert within the field. It is not perfect, but overall, this book represents an excellent primer for the complete novice, or for instructors who wish to introduce the topic of epigenetics to students with little or no knowledge of the topic.

## Conflict of interest statement

The author declares that the research was conducted in the absence of any commercial or financial relationships that could be construed as a potential conflict of interest.
